# Bioaccumulation and biomagnification of short and medium chain polychlorinated paraffins in different species of fish from Liaodong Bay, North China

**DOI:** 10.1038/s41598-017-06148-5

**Published:** 2017-09-07

**Authors:** Huiting Huang, Lirong Gao, Dan Xia, Lin Qiao

**Affiliations:** 1 State Key Laboratory of Environmental Chemistry and Ecotoxicology, Research Center for Eco-Environmental Sciences, Chinese Academy of Sciences, No. 18 Shuangqing Road, Haidian District Beijing, 100085 China; 2 University of Chinese Academy of Sciences, No. 19A Yuquan Road, Shijingshan District Beijing, 100049 China

## Abstract

Chlorinated paraffins (CPs) are highly complex technical mixtures, and the short chain chlorinated paraffins (SCCPs) are classed as persistent and have been included in the Stockholm Convention. However, there have been few studies of SCCPs and medium chain chlorinated paraffins (MCCPs) and their bioaccumulation and biomagnification in different species of fish. The present study investigated the levels, congener group profiles, bioaccumulation, and biomagnification of SCCPs and MCCPs in different species of fish from Liaodong Bay, North China. The ranges for the ΣSCCP and ΣMCCP concentrations were 376.3–8596 ng/g lipid weight (lw) and 22.37–5097 ng/g lw, respectively. The logarithms of bioaccumulation factors of ΣSCCPs ranged from 4.69 to 6.05, implying that SCCPs bioaccumulated in the fish. The trophic magnification factor of ΣSCCPs was 2.57, indicating that SCCPs could biomagnify in fish. Carbon chain length, the numbers of chlorine atoms, and octanol/water partition coefficients of the SCCPs and MCCPs might be important factors affecting the bioaccumulation of these chemicals in fish. The risk posed to human health by consumption of fish containing SCCPs was low. New SCCPs with nine carbons (C_9_) were detected in fish in this study.

## Introduction

Chlorinated paraffins (CPs) are polychlorinated n-alkanes with low volatility that have flame retardant and good electrical insulation properties. They are also inexpensive and widely used as flame retardants and plasticizers, and added to products such as paints, coatings, metal working fluids, and sealants^1–3^. Depending on their carbon chain length, CPs are classified into the following three categories: short chain chlorinated paraffins (SCCPs, C_10–13_), medium chain chlorinated paraffins (MCCPs, C_14–17_), and long chain chlorinated paraffins (LCCPs, C_18–30_)^4, 5^. The degree of chlorination of CPs is usually between 30 and 70% by weight^4^. Because of their toxicity^6–8^, persistence^9–13^, and potential to undergo long-range transport^14, 15^ and bioaccumulate^16, 17^, SCCPs have been included on the list of persistent organic pollutants in the Stockholm Convention.

China began to produce CPs in the late 1950s, and the total yield has continuously increased since then. In 2003, the annual production of SCCPs in China was approximately 150 kilotonnes^18^. This increased to 600,000 tons in 2007^19^, and 1000,000 tons in 2009^16^. China has become the main producer, user, and exporter of CPs in the world. Release of CPs can occur during their production, storage, transportation, and use, and during disposal of CPs and products that contain them^20^. SCCPs and MCCPs are found in all environmental matrices in China, including air^21^, water^22^, sediments^23^, soil^24^, biota^16^, terrestrial bird species^2^, mollusks^25^, and marine mammals^18^.

Fish are known to accumulate hydrophobic organochlorine pollutants in the environment^26^. Because food, and especially fish, is an important route of uptake of CP contaminants^27^, it is necessary to assess the levels of pollution in fish. However, limited data are available on SCCP and MCCP concentrations in fish^28–32^. Zhou *et al*. recently investigated the total CP concentrations for one fish species from the Yangtze River Delta, but did not study CP homologue group patterns^33^. Saborido Basconcillo *et al*. discussed the atmospheric sources or urban/industrial sources based on SCCP and MCCP homologue group profiles in top predatory fish across Canada^34^, but did not investigate bioaccumulation and biomagnification in fish. Zeng *et al*. and Ma *et al*. have conducted studies on bioaccumulation and biomagnification of SCCPs in food webs^16, 35^, but these studies were limited to SCCPs other than MCCPs. Furthermore, a study from Lake Ontario and Lake Michigan showed that SCCPs and MCCPs could bioaccumulate and biomagnify in food webs^17^, but did not discuss the effect of carbon chain length, chlorine atoms, octanol–water partition coefficients (K_ow_), and other factors on bioaccumulation and biomagnification.

To study the bioaccumulation and biomagnification of SCCPs and MCCPs in fish, different species of fish from Liaodong Bay, North China were collected. SCCPs and MCCPs were analyzed using comprehensive two-dimensional gas chromatography-electron-capture negative ionization-high resolution time-of-flight mass spectrometry (GC × GC-ECNI-HRTOF-MS). The three major objectives of the present study were as follows: (1) to investigate the levels and congener group profiles of SCCPs and MCCPs in different species of fish in this area; (2) to study bioaccumulation and biomagnification of SCCPs and MCCPs in the fish; and (3) to assess the human health risk of SCCPs and MCCPs in the fish.

## Results and Discussion

### Levels of SCCP and MCCP in fish

SCCPs and MCCPs were detected in all the fish samples collected from Liaodong Bay (Table 1). SCCP concentrations in the fish ranged from 67.80 to 1831 ng/g wet weight (ww), with an average of 427.8 ng/g ww. The MCCP concentrations were between 4.03 and 1022 ng/g ww with a mean value of 132.2 ng/g ww. The lipid weight (lw) for SCCPs ranged from 376.3 to 8596 ng/g lw (mean 2131 ng/g), and for MCCPs ranged from 22.37 to 5097 ng/g lw (mean 654 ng/g).

**Table 1:** SCCP and MCCP concentrations in different species of fish.

Fish species	SCCP concentrations (ng/g ww)	SCCP concentrations (ng/g lw)	MCCP concentrations (ng/g ww)	MCCP concentrations (ng/g lw)
Bastard halibut	1831 ± 586*	8596 ± 2751	150.5 ± 51.1	706.5 ± 240.2
Turbot	808.8 ± 347.8	4035 ± 1735	1022 ± 449	5097 ± 2242
Ray	166.1 ± 69.8	2233 ± 938	8.11 ± 3.32	109.0 ± 44.6
Navodon septentrionalis	390.0 ± 136.5	1750 ± 612	83.76 ± 26.80	375.9 ± 120.2
Yellow croaker	328.1 ± 134.5	1383 ± 567	13.09 ± 5.62	55.19 ± 23.73
Bass	195.3 ± 85.9	974.5 ± 428.7	4.92 ± 2.06	24.57 ± 10.31
Capelin	231.3 ± 83.3	863.0 ± 310.6	8.11 ± 3.08	30.26 ± 11.49
Spanish mackerel	155.7 ± 60.7	660.2 ± 257.4	12.72 ± 5.34	53.92 ± 22.64
Abalone	103.7 ± 43.6	440.2 ± 184.8	14.96 ± 5.83	63.48 ± 24.75
Cod	67.80 ± 30.51	376.3 ± 169.3	4.03 ± 1.65	22.37 ± 9.17

^*^Values shown are mean concentrations ± standard deviation.

SCCP concentrations and MCCP concentrations in the present study were much more than those found in earlier studies for SCCPs (11–70 ng/g ww) and MCCPs (7–47 ng/g ww) in cod liver samples from the European Arctic^31^. In addition, the concentrations were more than 10 times higher than the SCCP concentrations (49–820 ng/g lw) and MCCP concentrations (6.2–320 ng/g lw) found in human milk-fat samples from the UK^36^. The present concentrations were also much higher than SCCP concentrations (19–286 ng/g ww) and MCCP concentrations (25–260 ng/g ww) in fish from the North Sea and Baltic Sea^29^. Similar CP concentrations (7000 ng/g lw) have been found in eels from rice fields in the Yangtze River Delta, China^33^. This comparison of results clearly shows that the CP concentrations measured to date in fish have been higher in China than in any other country in the world, and this emphasizes the importance of further studies of CPs in the environment in China.

**Figure 1:**
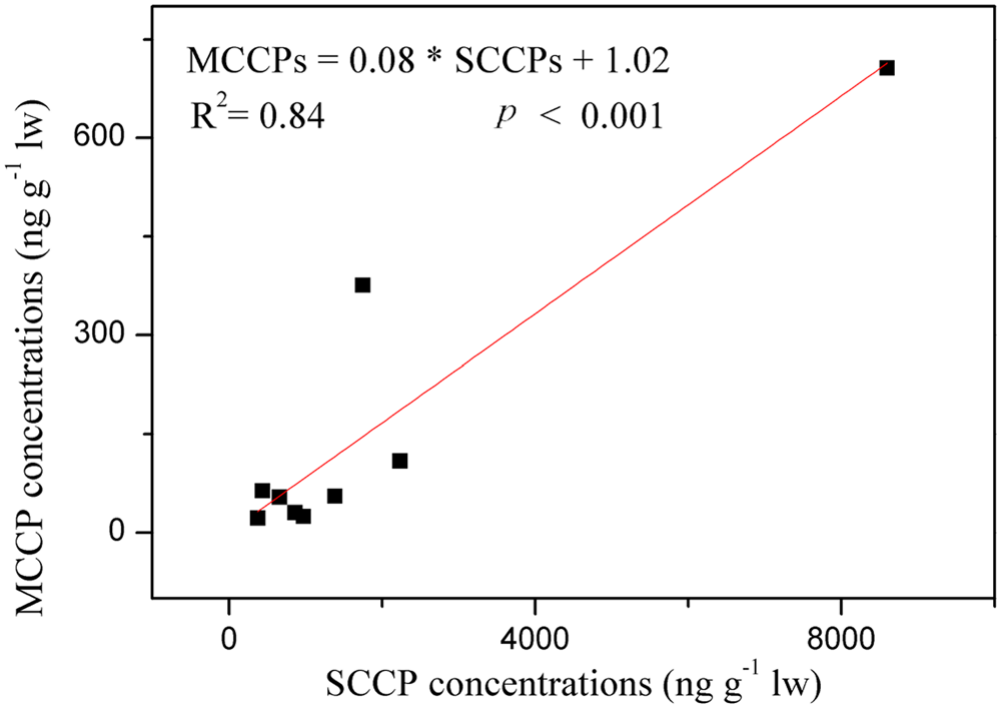
Correlation between ∑SCCPs and ∑MCCPs in the fish.

Among the fish species, lipid normalized SCCP concentrations were the highest in bastard halibut (8596 ng/g lw), followed by turbot (4035 ng/g lw). The lowest level was observed in cod (376.3 ng/g lw), and the second lowest level was in abalone (440.2 ng/g lw). For MCCP, lipid normalized concentrations were the highest in the turbot (1022 ng/g lw), followed by bastard halibut (706.5 ng/g lw). Again, the lowest level was in cod (22.37 ng/g lw), and the second lowest in abalone (63.48 ng/g lw). In all the fish except turbot, the SCCP concentrations were higher than the MCCP concentrations. Turbot could have different absorption rates of SCCPs and MCCPs compared with the other fish species. Except for turbot, there was a significant positive relationship between the concentrations of SCCPs and those of MCCPs in all the fish species (R^2^ = 0.84, *p* < 0.001, Fig. 1). This implies that these species of fish have similar uptake pathways and comparable net uptake rates of SCCPs and MCCPs^18, 37^.

### Congener and homologue group patterns

**Figure 2:**
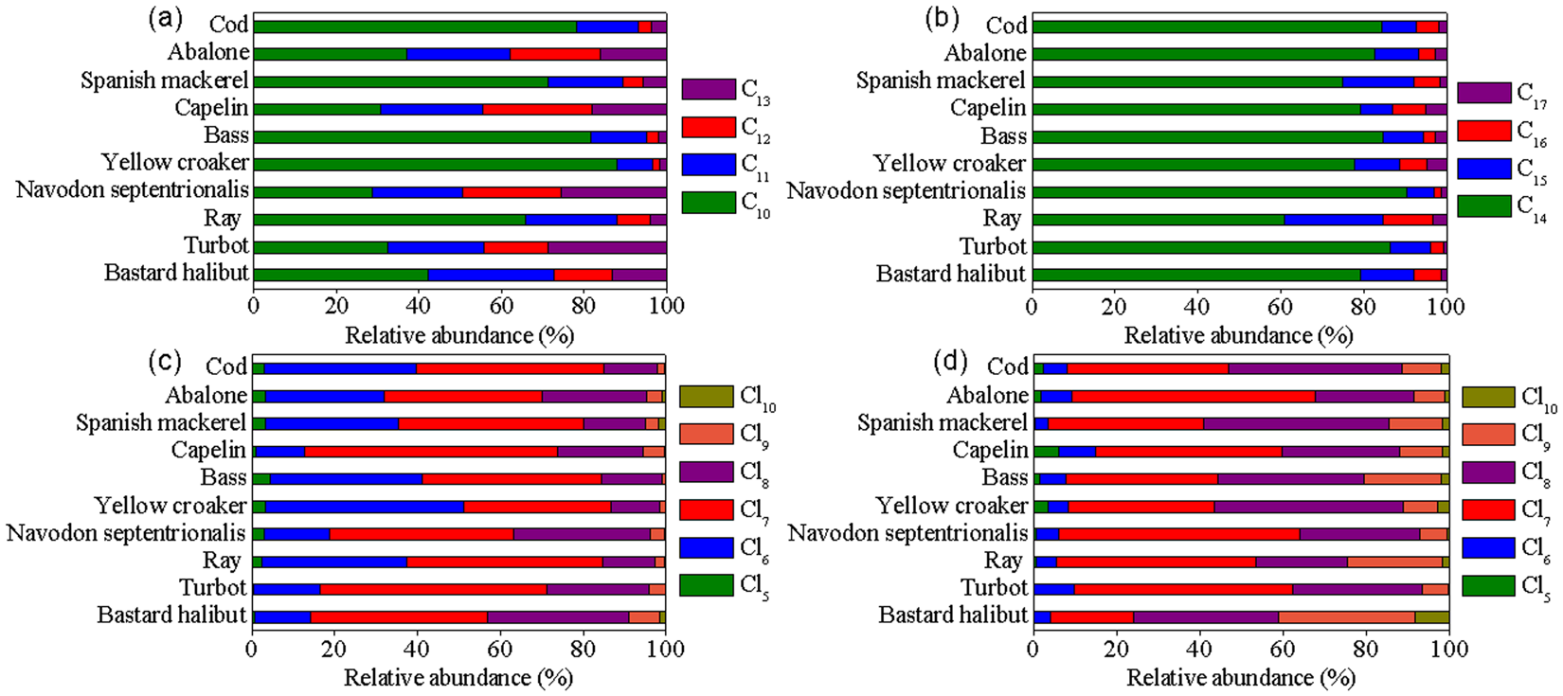
Congener group abundance profiles of SCCPs and MCCPs in the fish.

Congener distributions of SCCPs and MCCPs in the fish are shown in Fig. 2. For all fish species, C_10_ was the primary homologue group of SCCPs with an average contribution of about 55.6% of the total SCCPs (Fig. 2(a)). This is similar to earlier results, which showed a relatively higher abundance of C_10_ congeners than other congeners in fish from the North Sea^29^. For most of the fish species (bastard halibut, ray, yellow croaker, bass, Spanish mackerel, abalone and cod), congeners with 10 and 11 carbon atoms dominated the composition profiles with an average contribution of about 85.2% of the total SCCPs (Fig. 2(a)). Ma *et al*. found that C_10_ and C_11_ (82.3 ± 7.7%) were the most abundant groups in organisms (zooplankton, invertebrates, and fishes) from Liaodong Bay, China^16^. C_10_ and C_11_ homologue groups have been also found to predominate in the finless porpoise^18^ and terrestrial birds^2^. By contrast, for the other three fish species in this study (turbot, *Navodon septentrionalis*, and capelin), an almost equal abundance of SCCP homologue groups was observed and C_10_ and C_11_ accounted for 53.9% of the total SCCPs (Fig. 2(a)). This is similar to results for terrestrial bird species inhabiting an e-waste recycling site in Guangdong province, South China^2^. In the present study, the predominant chlorinated homologue group pattern for SCCPs in all the fish species was Cl_6_, Cl_7_, and Cl_8_. In total they added up to 93.6% of all SCCPs (Fig. 2(c)). Zeng *et al*. also found that C_11–12_ groups with 6–8 chlorines were the dominant congeners in fish from Gaobeidian Lake, China^35^. Although the dominant carbon chain lengths found by Zeng *et al*. (C_11–12_) were different from those in the present study (C_10–11_), the primary homologue group patterns (Cl_6–8_) were the same. This comparsion result might be because of the different pollution sources^34^. In the present study, congener patterns varied widely among the different species, and this could be caused by differences in transport and distribution in the environment as well as bioaccumulation and metabolization^29^. The most abundant homologue groups of SCCPs in the present study were generally C_10_Cl_6_ and C_10_Cl_7_ in all the fish species.

Congener distributions of MCCPs showed that C_14_ was the dominating homologue group in all the fish species, accounting for 60.7–96.5% of total MCCPs (Fig. 2(b)). C_15_ was the second most abundant group (6.7–24.0%), followed by C_16_ and C_17_. The distribution of the homologue groups of MCCPs in present study was consistent with that in biota from the European Arctic^31^, and in top predatory fish from nine freshwater bodies across Canada^34^. MCCPs with between seven and nine chlorines (total contribution 90.1%) predominated in all fish samples (Fig. 2(d)), and C_14_Cl_7_ and C_14_Cl_8_ were the most abundant groups. A similar profile was observed in top predatory fish from Lake Huron, Lake Ontario, and the Saint Lawrence River^34^.

### Bioaccumulation

Bioaccumulation factors (BAFs) are derived from concentration data collected in the environment, and used to determine whether it is possible for a chemical to bioaccumulate^38^. If the BAF of the chemical is greater than 5000, it is considered bioaccumulative. In the present study, BAFs were calculated in the fish species from Liaodong Bay based on SCCP values measured in fish and water samples. The water data were obtained from a study^16^. The details for the calculation method and results are shown in the SI (Table S1). The log BAFs of ΣSCCPs for the different fish species ranged from 4.69 to 6.05 with an average value of 5.24, indicating that SCCPs bioaccumulated in the fish. The log BAFs were slightly higher than those found in fish from Liaodong Bay, China^16^ (range 4.7–5.6, mean 5.08), but were slightly lower than those reported in trout from Lake Ontario^17^ (range 5.2–6.4, mean 6.1). The highest and lowest log BAFs in the present study were found in bastard halibut and cod, respectively. The lipid content and trophic level (TL) of bastard halibut were 1.94% and 3.81, respectively. The lipid content and TL of cod were 0.71% and 3.56, respectively. Wan *et al*. found that lipid content and TL were dominant factors determining accumulation of ΣPCBs in fish^39^. Based on this, and the fact that SCCPs and PCBs have similar properties, it is likely that lipid content and TL are important factors that determine the accumulation of SCCPs. Therefore, TL and lipid content might influence the accumulation of SCCPs in fish.

The log BAFs of 48 SCCP congeners ranged from 2.14 to 7.43 (mean 4.95), with the highest value for C_13_Cl_8_ in bastard halibut and the lowest value for C_12_Cl_5_ in Navodon septentrionalis. The ranges of log BAFs were similar to those in an earlier study^16^, where th**e** log BAFs of SCCP congener groups for all organisms varied from 4.1 to 6.7 (average 5.1). In addition, they were similar to the range (4.1–7.5) in Lake Ontario for SCCP congeners that were detected in water and aquatic organisms^17^. Based on the average log BAFs for SCCP congener groups in the different fish species, three SCCP congeners (C_11_Cl_5_, C_12_Cl_5_, and C_13_Cl_5_) might not bioaccumulate in the fish. The log BAFs of C_11_Cl_5_, C_12_Cl_5_, and C_13_Cl_5_ were 3.03, 2.83, and 3.26, respectively. The low BAFs could be attributed to the low chlorination of these SCCPs, which would mean they would be easy to metabolize and eliminate compared with SCCPs with higher chlorination^32, 40^.

**Figure 3:**
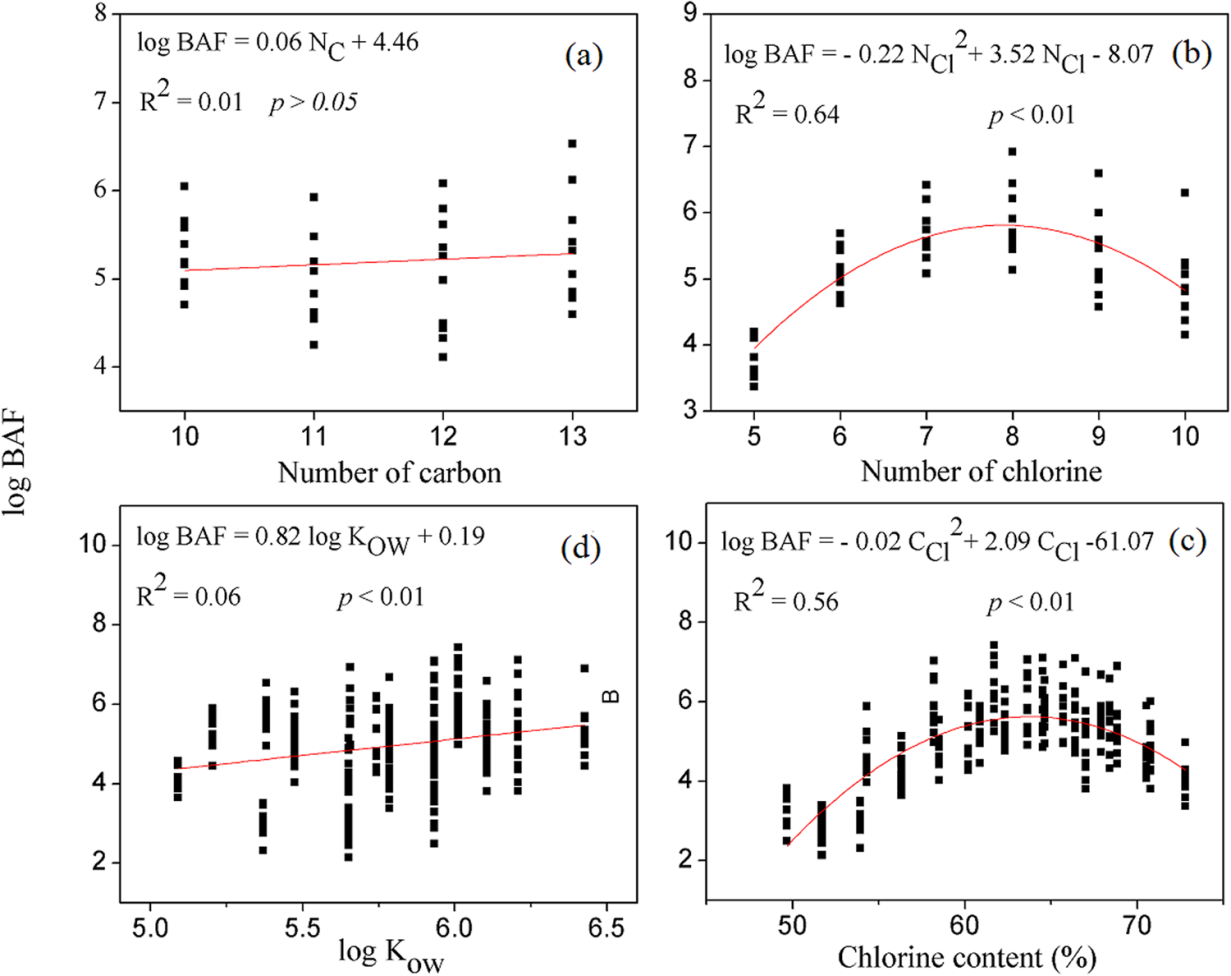
Relationships between log BAF of the SCCP congener groups and the number of carbon atoms, chlorine atoms, chlorine contents, and log K_ow_.

The log BAF values of the SCCP congener groups increased with increasing carbon chain length (Fig. 3(a)), although there was no significant linear relationship between them. This result is consistent with the conclusion of an earlier study of dietary exposure of juvenile rainbow trout, which found that the bioaccumulation potential of SCCP congeners generally increased with carbon chain length^12^. Another earlier study found a slight increasing trend for log BAFs with the number of carbon atoms (*p* > 0.05) in different fish species^35^. In addition, Ma *et al*. found a significant increasing trend between BAF values of SCCP congener groups and carbon chain length^16^. Therefore, as the carbon chain length increases, the bioaccumulation potential of SCCP congeners will increase.

A parabolic correlation was observed between log BAFs and the number of chlorine atoms (R^2^ = 0.64, *p* < 0.001), with the maximum value occurring at approximately eight chlorine atoms (Fig. 3(b)). Similar results were observed for the log BAF and chlorine contents (R^2^ = 0.56, *p* < 0.001) (Fig. 3(c)). These results are similar to those of Zhu *et al*.^40^ and Wang *et al*.^41^, who found a parabolic correlation between log BCFs and the number of chlorine atoms for PCB congeners. By contrast, Ma *et al*. and Zeng *et al*. observed a significant or non-significant linear relationship between log BAFs and the number of chlorine atoms^16, 35^. In the present study, the upward trend in the initial part of the parabolic curve could be attributed to the following: (1) a significant linear relationship between log K_ow_ and the number of chlorine atoms (R^2^ = 0.48, *p* < 0.001) (Fig. S1), and (2) for small molecules, the bioaccumulation potential (log BAF) increased as the hydrophobicity increased (log K_ow_). The downward trend in the latter part of the parabolic curve when the number of chlorine atoms was greater than eight could be attributed to the following: (1) the difficulty for highly chlorinated SCCP congeners (large molecules) to migrate across membranes, and (2) the relatively fast metabolic degradation of higher chlorinated SCCPs in fish compared with lower chlorinated congeners^41^.

The BAF values of the SCCP congener groups showed a significant linear increasing trend with increasing K_ow_ (Fig. 3(d)), indicating that K_ow_ might be a major factor governing congener specific bioaccumulation. Similar results have been reported by Zeng *et al*.^35^ and Ma *et al*.^16^. To a certain extent, the above results imply that carbon chain length, number of chlorine atoms, K_ow_ values, lipid content, TL, fish habit, and metabolization might be important factors determining the bioaccumulation of SCCP congeners in fish.

The bioaccumulation potential of MCCPs in the fish could be evaluated using BAF and K_ow_ values. In the present study, the MCCP concentrations in the water from Liaodong Bay were not available, and BAF values could not be calculated for the MCCPs. The current international protocol for persistent organic pollutants and management policies in Canada consider chemicals with a log K_ow_ > 5 as bioaccumulative^42^. Reported log K_ow_ values for MCCPs were in the range 6.83–8.96^43^. Therefore, MCCPs are considered as bioaccumulative, and this has been shown in other study^17, 44^.

### Biomagnification

To investigate biomagnification, we selected a number of aquatic species across multiple trophic levels and with predator–prey relationships. Samples were collected of invertebrates (jellyfish, Conch neptunea, clams, and Patinopecten yessoensis and mantis shrimp) and fish (bastard halibut, ray, Navodon septentrionalis, bass, and abalone). Stable isotopes of nitrogen are useful for assessing the TL of a marine species^16^. In the present study, TLs were determined based on stable nitrogen isotope ratios to investigate if biomagnification of SCCPs and MCCPs occurred in the organisms. The results (Fig. S2) showed that the TLs of the selected aquatic species ranged from 2.31 to 3.81. Trophic magnification factors (TMFs) were calculated as 10 to the power of the slope of the linear regression line between the logarithms of the concentrations (lw) of the CPs and the TLs (e.g. TMF = 10^b^ where b = the slope). The TMFs were used to estimate the magnitude of biomagnification of CPs in the organisms. The above ten species organism were included in the TMF determinations (see Table S2).

The calculated TMFs ranged from 0.39 to 11.47 for the SCCP congeners (24 congeners analyzed individually, Table S3). The TMFs of SCCP congener groups in this study were similar to or slightly higher than those (1.45–5.65) of SCCP congeners in the marine web in Liaodong Bay^16^, in the food web in Lake Ontario (0.47–1.5)^17^, and in Lake Michigan (0.41–2.4)^17^. The TMFs of C_10_Cl_7_ and C_10_Cl_8_ were 4.80 and 6.91, respectively (Table S3, *p* < 0.05). The TMFs of C_11_Cl_6_, C_11_Cl_7_, C_11_Cl_8_, and C_11_Cl_9_ were 3.96, 10.33, 11.47 and 8.32, respectively (Table S3, *p* < 0.05). The above TMFs were all greater than one and indicated biomagnification occurred in the organism^38, 45^. The specific TMFs for other homologue groups were not evaluated because of their weak linear relationships (*p* > 0.05). For the predominant carbon chains, the mean TMFs were 3.69 for C_10_ (*p* = 0.06), and 8.39 for C_11_ (*p* < 0.05), showing biomagnification of these compounds occurred. The TMF of ΣSCCPs was 2.57, indicating that biomagnification of SCCPs could occur in the fish.

The calculated TMFs of MCCP congeners ranged from 0.23 to 2.92 (Table S3). The TMFs in the present study were higher than those (0.06–0.36) found for MCCP congeners in a food web in Lake Ontario^17^. For the predominant carbon chain length (C_14_), the mean TMF was 3.69 (*p* > 0.05). The TMF of the ΣMCCPs was 0.71 (R^2^ = 0.02, *p* > 0.05). Linear relationships (Table S3) between the logarithms of the concentrations of ΣMCCPs (lw) in the organisms and TLs were weak, with almost all the *r* ^2^ values smaller than 0.1 and all *p* values greater than 0.05. Therefore, MCCP biomagnification in the fish did not occur.

### Evaluation of the risk to human health

The risk evaluation for SCCPs was based on the following: (1) the World Health Organization (WHO) health guidelines for neoplastic effects (tumor formation) of 11 µg/kg bw/day; and (2) the International Programme on Chemical Safety (IPCS) tolerable daily intake for SCCPs of 100 µg/kg bw/day^46^. The estimated daily intake (EDI, ng/kg bw/day) was used to represent the daily intake of SCCP via fish consumption per person per day and was calculated as follows:

1 $${\rm{EDI}}=\frac{C\times CV}{BW}$$

where *C* is the average concentration of SCCPs in the fish (ng/g ww), *CV* is the quantity of fish consumed per person per day (g/person/day), and *BW* is the average mass of the consumer (set at 60 kg). In the Chinese population, the rate of fish consumption for the low fish consumption group was set at 11 g/person/day, and the rate of fish consumption for the high fish consumption group was set at 119 g/person/day^47^.

For the low and high consumption groups, the EDIs for consumption of all species of fish (Table 2) were lower than the WHO and IPCS guidelines. However, when the high consumption group ate bastard halibut, the EDI was 33% of the WHO health guideline (11 µg/kg bw/day), which means that the WHO guideline could be easily exceeded if a person consumes this kind of fish regularly. Therefore, consumers, especially those who eat fish regularly, should adjust their diet to reduce the risk of exceeding the WHO and IPCS guidelines. In addition, because of the similar physico-chemical properties and toxicity profiles of SCCPs and MCCPs, simultaneous exposure to SCCPs and MCCPs will increase the risk^45^.

**Table 2:** Estimated daily intake for SCCPs in fish tissue.

Fish species	EDI of SCCPs (ng/kg d)	
	Population eating less fish	Population eating more fish
Bastard halibut	335.7	3631
Turbot	148.3	1604
Ray	30.45	329.4
Navodon septentrionalis	71.50	773.5
Yellow croaker	60.16	650.8
Bass	35.81	387.4
Capelin	42.41	458.8
Spanish mackerel	28.55	308.9
Abalone	19.02	205.7
Cod	12.43	134.5

### Detection of new SCCPs with nine carbon atoms in the fish

CPs are extremely complex mixtures because there are many possible positions for chlorine atom substitution^48^. In these complex mixtures, many of the CP congeners have similar chromatographic retention characteristics and cannot be separated and identified using one-dimensional gas chromatography. The GC × GC-HRTOF-MS method^49^ used in this study has high resolution, high sensitivity, and high peak capacity, and could separate CPs in these complex mixtures. Previously, studies have focused on only the C_10–13_ SCCPs, and SCCPs with nine carbons (C_9_) have not been investigated. One study made reference to C_9_ congeners because they have similar mass-to-charge ratios to C_14_ congeners and cannot be separated from them using low resolution mass spectrometry^50^. Wyatt *et al*. pointed out that studies in rats and mice have shown SCCPs are potentially carcinogenic, while there is no evidence of carcinogenicity for MCCPs and LCCPs^6^. In addition, some studies have reported that the toxicities of CP congeners generally increase as the carbon chain length decreases^45, 50^. Therefore, it is important to study C_9_ congeners.

In the present study, standard SCCP (C_10–13_) and MCCP (C_14–17_) mixtures with different chlorine contents were used to establish linear calibration curves. Because these standards did not contain C_9_ compounds, a semi quantitative method was to describe the relative amounts of C_9_ congeners as the percentage ratio of relative abundance of each homologue over the total relative abundance. Because most of the C_9_ congeners were detected at very low concentrations, only two C_9_ congeners (C_9_Cl_6_ and C_9_Cl_7_) were determined. The relative amounts of these C_9_ congeners in all the fish ranged from 0.92% to 8.38% (Table 3). The relative amounts of C_9_ congeners were more than those of C_12_ or C_13_ congeners in half of the fish species (ray, yellow croaker, bass, Spanish mackerel and cod). Because C_9_ and C_10_ have similar characteristics, the percentage ratio of relative abundance of C_9_ over that of C_10_ was calculated. The results from this ranged from 1.46% to 14.05%. Therefore, C_9_ is important in risk assessments and an accurate method needs to be developed for its quantification.

**Table 3:** The percentage of relative peak area of each component accounting for total relative peak area in the fish.

Fish species	C_9_	C_10_	C_11_	C_12_	C_13_	C_9_/C_10_
Bastard halibut	5.58	39.73	28.81	13.32	12.56	14.05
Turbot	1.37	31.99	23.00	15.25	28.39	4.30
Ray	8.38	60.34	20.19	7.48	3.62	13.89
Navodon septentrionalis	0.42	28.58	21.86	23.60	25.54	1.46
Yellow croaker	2.15	86.09	8.54	1.51	1.71	2.50
Bass	6.05	76.60	12.67	2.68	1.99	7.90
Capelin	0.92	30.49	24.34	26.20	18.04	3.00
Spanish mackerel	6.98	66.21	17.01	4.40	5.39	10.54
Abalone	4.54	35.39	23.85	20.97	15.26	12.83
Cod	5.63	73.80	13.95	3.24	3.37	7.63

## Conclusion

The SCCP and MCCP levels in the fish from Liaodong Bay are higher than or comparable to those in other studies. The C_10_ and Cl_6–8_ SCCPs and C_14_ and Cl_7–9_ MCCPs are the primary homologue groups in all of the fish species. The log BAFs of the SCCPs indicate bioaccumulation of SCCPs occurs in the fish, except for three SCCP congeners (C_11_Cl_5_, C_12_Cl_5_, and C_13_Cl_5_). The properties of the SCCP congeners (e.g. carbon chain length, number of chlorine atoms, and K_ow_), lipid content, trophic level and habit of the fish, and metabolization might be important factors affecting the bioaccumulation of SCCP congeners in the fish. Based on the K_ow_ values of the MCCP congeners, the MCCPs are considered as bioaccumulative. For the predominant carbon chain, the mean TMFs are 3.69 for C_10_, and 8.39 for C_11_, showing biomagnification of these compounds occurs in the organism. The TMF of ΣSCCPs is 2.57, indicating that SCCPs also have biomagnification potential in fish. The results suggest the risk to humans posed by consumption of fish containing SCCPs is low. We detected new SCCPs (C_9_) in the fish samples. Further research is required for toxicology and risk assessments.

## Methods

### Sample collection and preparation

Liaodong Bay is one of the three bays forming the Bohai Gulf, the innermost gulf of the Yellow Sea, in northeast China. And it borders Liaoning province. Ten species of fish and five species of invertebrates were collected from Liaodong Bay, North China in July 2014. All samples were wrapped in aluminum foil and transported to the laboratory. The fish samples were weighed and their lengths measured (Table 4). Details for the invertebrate samples are listed in Table S4. All samples were freeze-dried, ground, homogenized, and stored in amber glass bottles at −20°C until required for extraction. The mass differences before and after freeze-drying the samples were used to calculate their water contents (Table 4). A 2-g dry sample was spiked with surrogate standard (2.5 ng of ^13^C_10_-*trans*-chlordane), and then extracted with dichloromethane (DCM)/*n*-hexane (1:1, *v*/*v*) in an accelerated solvent extraction apparatus (ASE350; Dionex, Sunnyvale, CA, USA). The extraction conditions were as follows: three extraction cycles at 100 °C and 1.03 × 10^4^ kPa, 5 min of heating, a 10 min static extraction, a flush volume of 60% and a N_2_ purge time of 60 s. The extract was evaporated to about 2 mL using a rotary evaporator (Heidolph, Schwabach, Germany). The lipid content was determined gravimetrically (Table 4), and the details for the calculation are given in the Supplementary information (SI).

**Table 4:** Details of fish samples collected from the Liaodong Bay, North China.

English names	Latin names	Number of samples	Weight (g)	Length (cm)	Trophic level	Water content (%)	Lipidcontent(%)
Capelin	*mallotusvillosus*	23	12.10–15.60	9–12	3.33^a^	70.48^b^	9.19^c^
Yellow croaker	*Larimichthys polyactis*	15	46.51–68.80	16–20	3.62	74.50	6.97
Cod	*Gadus*	3	319.1–487.0	41–47	3.56	81.85	0.71
Turbot	*Scophthalmus maximus*	1	891.3	35	3.87	79.77	0.91
Bastard halibut	*Cleisthenes herzensteini*	5	101.1–118.1	23–25	3.81	78.28	1.94
Navodon septentrionalis	*Thamnaconus modestus*	3	199.9–378.2	24–31	3.41	77.60	0.54
Bass	*Cantharus*	2	589.5–1038	38–42	3.18	79.35	2.94
Abalone	*Abalone*	10	24.48–62.32	5–10	2.98	76.27	0.70
Spanish mackerel	*Spanish lacertus*	3	320.8–488.4	32–37	3.65	75.38	4.19
Ray	*Rajiformes*	8	102.3–123.5	30–33	3.53	92.50	0.84

^a,b,c^are arithmetic mean value.

The extracts were primarily cleaned up using gel permeation chromatography to remove sulfur containing compounds, lipids, and other interfering compounds (e.g. toxaphenes). The sample was added to the column, and then the column was cleaned with 70 mL of DCM/n-hexane (1:1 *v*/*v*), which was discarded. The sample was eluted with 130 mL of DCM/n-hexane (1:1 *v*/*v*), which was collected for further cleanup. The extract was then reduced to about 1 mL under reduced pressure. A multi-layer silica gel column was prepared by packing with 3 g of Florisil, 2 g of activated silica gel, 5 g of acidified silica gel (44% mass fraction sulfuric acid), and 5 g of anhydrous Na_2_SO_4_ from bottom to top. The multilayer column was rinsed with 50 mL of n-hexane before use. Then the sample was added and eluted with 40 mL of n-hexane, which was discarded. Afterwards, the column was eluted with 100 mL of DCM/n-hexane (1:1 *v*/*v*), which was collected for analysis of CPs. The eluate was concentrated to about 5 mL using a rotary evaporator. The fraction containing SCCPs and MCCPs was reduced to about 0.5 mL and transferred to a vial. The solution in the vial was further concentrated to near dryness under a gentle stream of N_2_. The solvent was replaced with 50 µL of cyclohexane. Before analysis, 2.5 ng of ɛ-hexachlorocyclohexane (ε-HCH) was added to the vial as an injection internal standard.

### Instrumentation and quantification

The GC × GC-ECNI-HRTOF-MS analyses were conducted using an Agilent 7890 A GC (Agilent Technologies, Santa Clara, CA, USA) fitted with a ZX2004 loop cryogenic modulator (Zoex corporation, Houston, TX, USA) interfaced with a high resolution time-of-flight MS (Tofwerk, Thun, Switzerland) operated in ECNI mode. For all fish samples, SCCPs and MCCPs analyses were carried on a GC × GC-ENCI-HRTOF-MS instrument. The first-dimension column was an Agilent DB-5 (5% diphenyl, 95% dimethyl polysiloxane; 30 m × 0.25-mm inner diameter (i.d.), 0.25-μm film thickness). The second-dimension column was a SGE BPX-50 (50% diphenyl, 50% dimethyl arylene polysiloxane; 1 m × 0.10-mm i.d., 0.10-μm film thickness). The initial GC oven temperature was 140 °C for 1 min, and then increased at 10 °C/min to 200 °C, and finally increased at 1.5 °C/min to 310 °C, and maintained at 310 °C for 5 min.

Injections were performed in splitless mode with an injection volume of 1.0 μL and an inlet temperature of 280 °C. The carrier gas flow rate (helium, 99.999% pure) was constant at 1 mL/min. Methane was used as the ECNI ionization agent with a flow rate of 2 mL/min. The electron energy was 125 eV and the emission current was 0.1 mA. The ion source and transfer line temperatures were 200 °C and 280 °C, respectively. The modulation period was 8 s. The hot gas duration time was 300 ms. The modulator hot gas temperature was 350 °C. The data acquisition speed was 100 Hz. This instrument had a mass resolution of 5000 (full width at half maximum) and a mass precision of 5 ppm or 0.002 u, using perfluoroperhydrophenanthrene for mass calibration. GC × GC data were processed using GC Image^®^ R2.5 Software (GC Image, Lincoln, NE, USA).

The two most abundant [M-Cl]^−^ ions were detected in full scan mode as quantitative and qualitative ions. The most abundant [M-Cl]^−^ ion was used as a quantification ion and the next most abundant ion was used as a qualification ion^50^. The quantification of SCCP congeners and MCCP congeners was conducted based on an established technique^48^. The quantification method has been reported in another study^49^ and was mainly dependent on linear correlation between the total response factors for CP standard mixtures and their chlorine content. In total, 48 SCCP (C_10–13_Cl_5–10_) and MCCP (C_14–17_Cl_5–10_) congeners were analyzed in the samples in this study. Detailed information on the chemicals can be found in the SI.

### Quality assurance and quality control

To eliminate background contamination, all glassware was heated to 200 °C, and thoroughly rinsed with methanol, acetone, and dichloromethane in succession. The results for three procedural blanks indicated that the concentrations of both SCCPs and MCCPs in the blanks were less than 5% of those found in the fish samples. Therefore, the final concentrations of SCCPs and MCCPs reported in this study were not blank corrected. The method detection limit (MDL), which was defined as the average CP contents in the blanks plus three times the standard deviation, was 9.4 ng/g for the SCCPs and 7.0 ng/g for the MCCPs in the fish. The recovery was calculated by dividing the ratio of the surrogate standard (^13^C_10_-*trans*-chlordane) and injection internal standard (ε-HCH) in each sample by the ratio of ^13^C_10_-*trans*-chlordane and ε-HCH in the appropriate standard solution. The surrogate recoveries of ^13^C_10_-*trans*-chlordane in all the fish samples ranged from 61.0% to 92.6%. Two of ten species of fish were randomly selected for parallel experiments. The relative standard deviation obtained after repeating the analysis of each sample seven times was less than 15%. Atmospheric nitrogen was used as δ^15^N standard. The laboratory working standard was STD-27 (δ^15^N_air_ = 7.0 ± 0.15‰). Replicate measurements of STD-27 gave a measurement error of 0.15‰ for stable nitrogen isotope measurements.

### Ethic Statements

No experiment on live vertebrates and higher invertebrates was included in this study. The study was carried out in compliance with relevant laws, guidelines, and regulations of China and under a permit issued by the Research Center for Eco-Environmental Sciences, Chinese Academy of Sciences.

## Electronic supplementary material

Supplementary Information
