# Supplementary material for: Bioaccumulation and biomagnification of short and medium chain polychlorinated paraffins in different species of fish from Liaodong Bay, North China
Source: Sci Rep. 2017 Sep 7;7:10749. doi: 10.1038/s41598-017-06148-5 (PMC5589728; doi:10.1038/s41598-017-06148-5)
Supplement: Supplementary file 1 — Supplementary Information [file 41598_2017_6148_MOESM1_ESM.pdf]

Bioaccumulation and biomagnification of short-and medium-chain polychlorinated paraffins in different species of fish from Liaodong Bay, North China

Huiting Huang [a,b], Lirong Gao [a,b *], Dan Xia [a,b], Lin Qiao [a,b]

[a] State Key Laboratory of Environmental Chemistry and Ecotoxicology, Research Center for Eco-Environmental Sciences, Chinese Academy of Sciences, No. 18 Shuangqing Road, Haidian District, Beijing 100085, China

[b] University of Chinese Academy of Sciences, No. 19A Yuquan Road, Shijingshan District, Beijing 100049, China

Journal: ***Scientific Reports***

Corresponding author: Lirong Gao

*E-mail address: gaolr@rcees.ac.cn

Phone number: +86 10-62849356

Fax number: +86 10-62849172

Chemicals and materials

Pesticide analysis grade n-hexane, acetone, and dichloromethane (DCM) were purchased from J.T. Baker (Phillipsburg, NJ, USA). Anhydrous Na_2SO_4 was baked at 660 ℃ for 6.5 h before use. Florisil was activated at 550 ℃ for 12 h. The silica gel was activated at 550 ℃ for 6.5 h. Acidified silica gel was prepared by adding 43 mL of 98% H_2SO_4 to 100 g of activated silica gel (44 % *w*/*w*). Three SCCP technical mixtures (100 ng/μL in cyclohexane) with chlorine contents of 51.0 %, 55.5 %, and 63.0 %, and three MCCPs mixtures (100 ng/μL in cyclohexane) with chlorine contents of 42.0 %, 52.0 %, and 57.0 %, were obtained from Dr. Ehrenstorfer GmbH (Augsburg, Germany). An internal standard of $^{13}C_{10}$-*trans*-chlordane (100 μg/mL in nonane, 99 % purity) was purchased from Cambridge Isotope Laboratories (Andover, MA, USA). A recovery standard of ε-Hexachlorocyclohexane (ε-HCH) was obtained from Dr. Ehrenstorfer GmbH (Augsburg, Germany).

Calculation of the lipid content

Lipid content was determined gravimetrically using a plough-shaped bottle. The bottle was weighed recorded as m_1 before use, and then the extract was transferred to the bottle. The extract was evaporated to about 2 mL using a rotary evaporator (Heidolph, Schwabach, Germany). The bottle should be placed over 24 h unless the weight was constant, and then was weighed recorded as m_2. 'm' in the equations 1 is the weight of each sample. Lipid content was calculated using Eq. (1) as follows:

$$\text{Lipid content (\%)} = \frac{m_1 - m_2}{m} \times 100 \quad (1)$$

Calculation of bioaccumulation factors (BAFs) for individual SCCP congener group

According to the method of Ma et al. [1], BAFs were calculated using Eq. (2) as follows:

$$\text{BAF} = \frac{C_{\text{i-fish}}}{C_{\text{i-water}}} \quad (2)$$

where $C_{\text{i-fish}}$ and $C_{\text{i-water}}$ represent the SCCP concentrations in fish (ng/g lw) and water (ng/L), respectively.

According to our quantification procedures and the method of Ma et al. [1], the BAFs of individual SCCP congener group were calculated using Eq. (3) as follows:

$$\text{BAF}_{ci} = \frac{\sum \text{SCCPs}_{(\text{fish})} \times X_{Fci}}{\sum \text{SCCPs}_{(\text{water})} \times X_{Wci}} \quad (3)$$

where X_{Fci} and X_{Wci} represent the relative abundance of that formula group in fish and seawater,

respectively.

Nitrogen isotope analysis to determine the trophic level

Stable nitrogen isotope ratios were measured in the samples collected in this study, and the results were used to determine the trophic levels. Three samples for each species were freeze-dried and then ground to a powder. The stable nitrogen isotope composition for each sample was determined using an automatic elemental analyzer (Flash EA1112, Thermo Fisher Scientific, America) connected to a Thermo Finnigan Delta Plus Advantage isotope ratio mass spectrometer (Thermo Fisher Scientific). The results are expressed in standard δ notation, calculated using Eq. (4) [2] as follows:

$$\delta^{15}N = \left(\frac{{}^{15}N/{}^{14}N_{sample}}{{}^{15}N/{}^{14}N_{standard}} - 1\right) \times 1000 \quad (4)$$

The trophic level for each species was calculated using Eq. (5) as follows:

$$TL = \frac{\delta^{15}N_{sample} - \delta^{15}N_{baseline}}{\Delta\delta^{15}N} + 2 \quad (5)$$

where ${}^{15}N/{}^{14}N_{standard}$ values were based on atmospheric nitrogen (air), TL is the trophic level of the fish, $\delta^{15}N_{baseline}$ is the nitrogen isotope ratio of a primary consumer in the marine environment, and $\Delta\delta^{15}N$ is the trophic enrichment factor (generally 3.4 ‰) [3]. We used the nitrogen isotope ratio found in the adductor muscle of the primary consumer *Chlamys farreri*, 5.84 ‰, as the baseline ratio.

Statistical analyses

Data analysis was performed using Origin Pro 8.0 software (OriginLab, Northampton, MA) and statistical analysis was performed using IBM SPSS Statistics 22.0 (IBM Corp., Armonk, NY). Before analysis, a normality test was performed with the raw data. Correlations between log concentrations of CP (on a lipid weight basis) and the TLs of organisms were examined by Spearman’s rank correlation test, and when the value of p was below 0.05, the linear regression was regarded as significant.

Table

Table S1. Log BAFs of SCCP congener groups and ∑SCCP in the fish.

Congeners	Bastard halibut	Turbot	Ray	Navodon septentrionalis	Yellow croaker	Bass	Capelin	Spanish mackerel	Abalone	Cod
$C_{10}Cl_5$	4.49*	3.86	4.53	4.56	4.47	4.46	3.65	4.16	3.96	3.87
$C_{10}Cl_6$	5.89	5.68	5.82	5.18	5.80	5.50	4.45	5.23	4.95	5.09
$C_{10}Cl_7$	6.54	6.09	6.09	5.66	5.89	5.79	5.57	5.58	4.95	5.37
$C_{10}Cl_8$	6.68	5.89	5.61	5.44	5.81	5.72	5.13	5.58	4.73	5.24
$C_{10}Cl_9$	5.90	4.86	4.60	4.07	4.69	4.73	4.29	4.30	4.20	4.38
$C_{10}Cl_{10}$	4.98	4.13	4.16	3.96	3.38	3.86	4.20	4.28	4.17	3.59
$C_{11}Cl_5$	3.50	3.17	3.46	2.95	3.13	3.08	2.88	3.01	2.76	2.31
$C_{11}Cl_6$	5.54	4.85	5.06	4.96	4.42	4.59	4.85	4.63	4.51	4.03
$C_{11}Cl_7$	6.31	6.00	5.69	5.41	4.94	5.03	5.23	4.95	4.79	4.77
$C_{11}Cl_8$	6.93	6.39	5.89	6.10	5.55	5.49	5.63	4.95	5.52	4.98
$C_{11}Cl_9$	6.58	5.91	5.72	5.51	5.12	4.32	5.08	5.48	5.12	4.66
$C_{11}Cl_{10}$	6.01	4.69	5.25	4.52	4.70	4.26	3.80	5.44	4.90	4.30
$C_{12}Cl_5$	3.19	3.39	3.14	2.14	2.78	2.63	2.91	2.44	3.06	2.58
$C_{12}Cl_6$	5.14	4.84	4.50	4.90	3.94	4.00	4.28	3.78	4.15	3.86
$C_{12}Cl_7$	6.20	6.15	5.37	5.85	4.41	4.69	5.88	4.82	5.39	4.28
$C_{12}Cl_8$	7.06	6.72	6.33	6.61	5.20	5.36	5.85	5.26	5.78	4.91
$C_{12}Cl_9$	7.10	6.26	5.91	6.20	5.32	4.90	5.79	5.02	5.48	4.71
$C_{12}Cl_{10}$	6.90	5.34	5.69	5.24	5.07	4.44	4.70	5.64	5.24	4.99
$C_{13}Cl_5$	2.97	3.69	2.99	3.82	3.68	3.29	3.28	2.49	3.55	2.87
$C_{13}Cl_6$	5.25	5.88	4.61	5.04	4.42	3.97	4.48	4.21	4.40	4.36
$C_{13}Cl_7$	6.64	7.03	5.88	6.55	5.22	5.23	5.91	5.47	5.64	4.97
$C_{13}Cl_8$	7.43	7.16	6.07	6.93	5.47	5.45	6.49	5.82	6.20	5.25
$C_{13}Cl_9$	7.11	6.76	5.80	6.18	5.39	5.23	6.29	5.32	5.38	4.86
$C_{13}Cl_{10}$	6.75	5.77	5.17	5.50	4.47	3.81	5.34	4.03	4.71	4.35
$\sum C_{10}$	6.05	5.61	5.66	5.19	5.58	5.39	4.91	5.16	4.70	4.96
$\sum C_{11}$	5.92	5.48	5.20	5.09	4.59	4.62	4.83	4.59	4.55	4.25
$\sum C_{12}$	6.08	5.79	5.26	5.62	4.33	4.44	5.36	4.49	4.98	4.11
$\sum C_{13}$	6.53	6.54	5.42	6.12	4.85	4.79	5.67	5.05	5.32	4.60
$\sum Cl_5$	4.16	3.62	4.19	4.20	4.12	4.10	3.37	3.81	3.63	3.52
$\sum Cl_6$	5.68	5.43	5.51	5.06	5.44	5.18	4.63	4.95	4.72	4.76
$\sum Cl_7$	6.42	6.20	5.88	5.74	5.55	5.48	5.58	5.32	5.08	5.09
$\sum Cl_8$	6.92	6.45	5.90	6.21	5.67	5.60	5.70	5.45	5.50	5.14
$\sum Cl_9$	6.59	6.00	5.49	5.59	5.03	4.76	5.46	5.11	4.99	4.57
$\sum Cl_{10}$	6.30	5.07	5.19	4.86	4.59	4.16	4.58	5.25	4.81	4.37
∑SCCPs	6.05	5.72	5.46	5.36	5.25	5.10	5.05	4.93	4.76	4.69

*BAF was the average.

Table S2 Species used for the TMF determinations.

Category	Species	Number of individual species
Invertebrate	Jellyfish	3
	Conch neptunea	10
	Clam	33
	Patinopecten yessoensis	20
	Mantis shrimp	12
fish	Abalone	10
	Bass	2
	Navodon septentrionalis	3
	Ray	8
	Bastard halibut	5

Table S3. Slope and *p* value of regression analysis between log concentrations (lw) and trophic levels, and trophic magnification factors of SCCP congener groups and ∑SCCPs as well as MCCP congener groups and ∑MCCPs in the invertebrate-fish.

**Congeners**	**SCCPs**				**Congeners**	**MCCPs**			
	slope	r^2	*p* value	TMF		slope	r^2	*p* value	TMF
$C_{10}Cl_5$	0.22	0.04	0.59	1.65	$C_{14}Cl_5$	0.47	0.15	0.26	2.92
$C_{10}Cl_6$	0.33	0.16	0.26	2.12	$C_{14}Cl_6$	0.07	0.00	0.87	1.17
$C_{10}Cl_7$	0.68	0.47	**0.03**	4.80	$C_{14}Cl_7$	-0.05	0.00	0.90	0.89
$C_{10}Cl_8$	0.84	0.55	**0.02**	6.91	$C_{14}Cl_8$	-0.14	0.01	0.77	0.73
$C_{10}Cl_9$	0.65	0.35	0.07	4.47	$C_{14}Cl_9$	0.01	0.00	0.97	1.03
$C_{10}Cl_{10}$	0.29	0.17	0.24	1.96	$C_{14}Cl_{10}$	0.05	0.00	0.92	1.11
$C_{11}Cl_5$	0.41	0.34	0.08	2.59	$C_{15}Cl_5$	0.15	0.02	0.71	1.42
$C_{11}Cl_6$	0.60	0.59	**0.01**	3.96	$C_{15}Cl_6$	-0.16	0.04	0.60	0.69
$C_{11}Cl_7$	1.01	0.75	**0.00**	10.33	$C_{15}Cl_7$	-0.32	0.07	0.48	0.48
$C_{11}Cl_8$	1.06	0.67	**0.00**	11.47	$C_{15}Cl_8$	-0.35	0.05	0.53	0.44
$C_{11}Cl_9$	0.92	0.46	**0.03**	8.32	$C_{15}Cl_9$	-0.48	0.09	0.41	0.33
$C_{11}Cl_{10}$	0.20	0.04	0.60	1.59	$C_{15}Cl_{10}$	-0.64	0.14	0.29	0.23
$C_{12}Cl_5$	-0.27	0.09	0.40	0.54	$C_{16}Cl_5$	0.38	0.12	0.33	2.38
$C_{12}Cl_6$	0.18	0.05	0.54	1.50	$C_{16}Cl_6$	-0.28	0.07	0.47	0.52
$C_{12}Cl_7$	0.12	0.02	0.74	1.32	$C_{16}Cl_7$	-0.37	0.08	0.43	0.42
$C_{12}Cl_8$	0.41	0.14	0.29	2.60	$C_{16}Cl_8$	-0.35	0.06	0.51	0.45
$C_{12}Cl_9$	0.51	0.14	0.29	3.23	$C_{16}Cl_9$	-0.60	0.11	0.34	0.25
$C_{12}Cl_{10}$	0.39	0.08	0.43	2.46	$C_{16}Cl_{10}$	-0.38	0.04	0.60	0.42
$C_{13}Cl_5$	-0.41	0.18	0.22	0.39	$C_{17}Cl_5$	0.32	0..05	0.55	2.10
$C_{13}Cl_6$	-0.37	0.09	0.39	0.43	$C_{17}Cl_6$	-0.11	0.01	0.75	0.78
$C_{13}Cl_7$	-0.21	0.03	0.61	0.61	$C_{17}Cl_7$	-0.03	0.00	0.95	0.93
$C_{13}Cl_8$	-0.09	0.00	0.85	0.80	$C_{17}Cl_8$	-0.29	0.05	0.55	0.51
$C_{13}Cl_9$	-0.01	0.00	0.98	0.97	$C_{17}Cl_9$	-0.45	0.12	0.32	0.35
$C_{13}Cl_{10}$	0.18	0.01	0.78	1.53	$C_{17}Cl_{10}$	-0.63	0.24	0.15	0.23

$\sum C_{10}$	0.57	0.39	0.06	3.69	$\sum C_{14}$	-0.04	0.00	0.92	0.92
$\sum C_{11}$	0.92	0.73	**0.00**	8.39	$\sum C_{15}$	-0.42	0.10	0.38	0.38
$\sum C_{12}$	0.29	0.08	0.44	1.94	$\sum C_{16}$	-0.40	0.08	0.44	0.40
$\sum C_{13}$	0.12	0.01	0.79	1.31	$\sum C_{17}$	-0.36	0.10	0.38	0.44
$\sum Cl_5$	0.23	0.05	0.53	1.70	$\sum Cl_5$	0.39	0.11	0.34	2.46
$\sum Cl_6$	0.36	0.27	0.12	2.29	$\sum Cl_6$	0.04	0.00	0.92	1.09
$\sum Cl_7$	0.42	0.27	0.12	2.61	$\sum Cl_7$	-0.10	0.01	0.80	0.79
$\sum Cl_8$	0.39	0.17	0.24	2.45	$\sum Cl_8$	-0.18	0.02	0.67	0.66
$\sum Cl_9$	0.30	0.06	0.49	1.99	$\sum Cl_9$	-0.20	0.02	0.66	0.63
$\sum Cl_{10}$	0.27	0.05	0.54	1.85	$\sum Cl_{10}$	-0.25	0.03	0.63	0.56
∑SCCPs	0.41	0.28	0.12	**2.57**	∑MCCPs	-0.15	0.02	0.72	0.71

Table S4. Details for invertebrate samples collected from Liaodong Bay, North China.

English names	Latin names	Length/ diameter (cm)	Trophic level	Lipid content (%)	SCCP concentrations (ng/g dw)	MCCP concentrations (ng/g dw)	SCCP concentrations (ng/g lw)	MCCP concentrations (ng/g lw)
Jellyfish	*Rhopilema*	-	2.31*	0.12*	2618*	447.7*	2622*	448.2*
Conch neptunea	*Neptunea cumingi Crosse*	6-8	2.61	3.94	622.7	62.41	648.2	64.97
Clam	*Clam*	4-6	2.99	7.19	958.9	171.8	1033	185.1
Patinopecten yessoensis	*Patinopecten yessoensis*	8-10	3.05	4.10	2250	322.1	2347	335.8
Mantis shrimp	*Oratosquilla oratoria*	10-13	3.78	11.67	2953	14.77	3343	16.72

* Values shown are arithmetic mean.

Figure

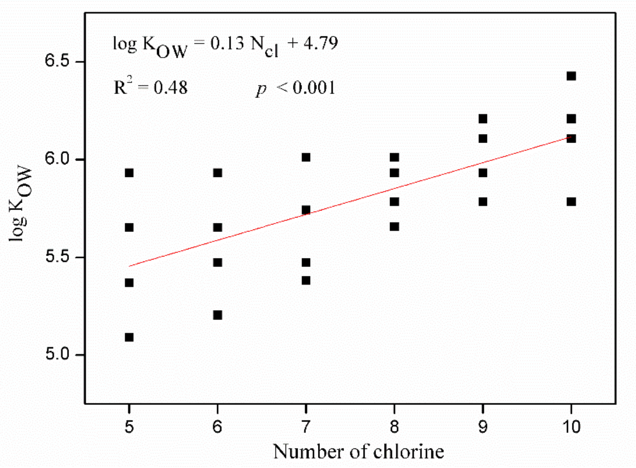

Figure S1. Correlation between log K_{OW} and number of chlorine atoms for SCCP congeners in the fish.

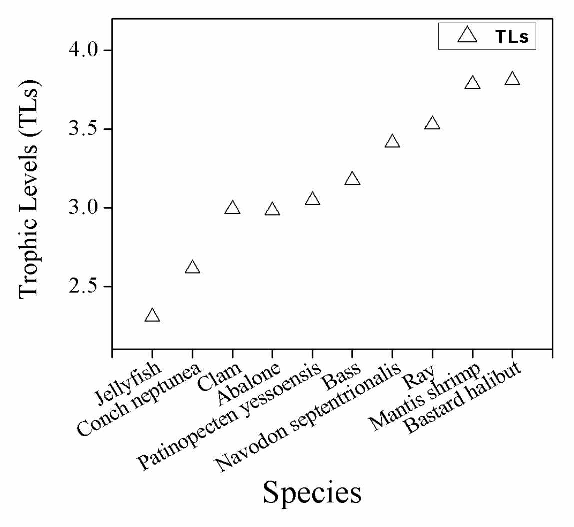

Figure S2. The mean trophic levels of the 10 species included in the TMF determinations.

References

1. Ma, X. et al. Bioaccumulation and trophic transfer of short chain chlorinated paraffins in a marine food web from Liaodong Bay, North China. *Environ. Sci. Technol.* **48**, 5964-5971 (2014).
2. Chang, N.-N., Shiao, J.-C., Gong, G.-C., Kao, S.-J. & Hsieh, C.-h. Stable isotope ratios reveal food source of benthic fish and crustaceans along a gradient of trophic status in the East China Sea. *Cont. Shelf Res.* **84**, 23-34 (2014).
3. Post, D.M. Using stable isotopes to estimate trophic position: Models, methods, and assumptions. *Ecology* **83**, 703-718 (2002).
